# The efficacy of pericapsular nerve group block for postoperative analgesia in patients undergoing hip surgery: A systematic review and meta-analysis of randomized controlled trials

**DOI:** 10.3389/fmed.2023.1084532

**Published:** 2023-02-24

**Authors:** Liang Yu, Xiaojuan Shen, He Liu

**Affiliations:** ^1^Department of Anesthesiology, Huzhou Key Laboratory of Basic Research and Clinical Translation for Neuromodulation, Huzhou Central Hospital, The Affiliated Huzhou Hospital, Zhejiang University School of Medicine, Affiliated Central Hospital Huzhou University, Huzhou, China; ^2^Huzhou Central Hospital, The Affiliated Huzhou Hospital, Zhejiang University School of Medicine, Affiliated Central Hospital Huzhou University, Huzhou, China

**Keywords:** pericapsular nerve group block, postoperative pain, regional, opioid consumption, anesthesia

## Abstract

**Background:**

As an optional regional anesthesia approach, pericapsular nerve group (PENG) block has been successfully utilized to manage pain for hip surgeries without affecting motor function. The present meta-analysis aimed to verify the efficacy of PENG block for postoperative analgesia in patients undergoing hip surgery.

**Methods:**

A total of 497 academic articles were identified after a systematic search in the databases of PubMed, Embase, Web of Science, and Cochrane Library up to 25 August 2022. The primary outcome was postoperative 24-h morphine consumption. Secondary outcomes included the time of the first request for rescue analgesia, static and dynamic pain scores 6 and 24 h after surgery, and incidence of postoperative nausea and vomiting (PONV). We calculated mean differences (MDs) with 95% confidence intervals (CIs) for postoperative 24-h morphine consumption, time of the first request for rescue analgesia, static and dynamic pain scores 6 and 24 h after surgery, and odds ratios (ORs) with 95% CIs for incidence of PONV. The chi-square test was used for heterogeneity analysis, and heterogeneity was assessed by *I*^2^. Statistical analysis was performed using Review Manager 5.4.

**Results:**

Numerous electronic databases were searched, and finally, nine studies were identified. There was no significant difference in morphine consumption during the postoperative 24 h [MD: −2.57, 95% CI: (−5.42, 0.27), *P* = 0.08] and the time of the first request for rescue analgesia [MD: 1.79, 95% CI: (−1.06, 4.64), *P* = 0.22] between the PENG block and control groups. PENG block did not reveal a significant difference in 6 h [MD: −0.17, 95% CI: (−0.92, 0.57), *P* = 0.65] [MD: −0.69, 95% CI: (−1.58, 0.21), *P* = 0.13] and 24 h [MD: −0.25, 95% CI: (−1.54, 1.05), *P* = 0.71], [MD: 0.05, 95% CI: (−0.84, 0.93), *P* = 0.91] static and dynamic pain scores compared with other nerve block methods. Moreover, the two groups have a similar risk of PONV (OR: 1.29, 95% CI = 0.53–3.10, *P* = 0.57).

**Conclusion:**

This review shows that PENG block can act as an alternative multimodal analgesia for hip surgery, and compared with the other kinds of nerve block, there was no significant difference in the postoperative analgesic effect of PENG block.

**Systematic review registration:**

Supplementary Datasheet 1, identifier: CRD 42022356496.

## 1. Introduction

Approximately 1.6 million people worldwide suffer a hip fracture each year, and this number is increasing by 25% every 10 years, as the population continues to grow (1). With the increasing aging of the world population, it is estimated that by 2050, the number of patients with hip fractures will reach 6.3 million (2). Early surgical treatment is recommended for hip fractures. Hip surgery can cause moderate to severe pain (3). In the process of rehabilitation, the restriction of exercise due to pain leads to adverse results. Multimodal analgesia in conjunction with nerve block techniques is often used to treat pain during the surgical period of hip fracture, as adequate pain management has been shown to reduce complications and promote postoperative activity. Pericapsular nerve group (PENG) block is a new blocking concept in which a local anesthetic is injected around the anterior hip capsule to block the nerves innervating the anterior hip capsule, mainly by blocking the hip branch of the femoral and para-occlusive nerves that travel between the anterior inferior iliac spine and the iliopubic ramus (4, 5). The anterior hip capsule is innervated by the femoral, accessory obturator, and obturator nerves (6). According to recent anatomical studies, the iliopubic ridge and the medial aspect of the inferior acetabulum are considered to be the relevant bony landmarks guiding the blocking of these three neuroarticular branches when performing the PENG block technique (5). These anatomical underpinnings led Girón-Arango et al. (4) to report, for the first time, PENG block, a new technique for selectively blocking the articular branches of the femoral, paracentral, and obturator nerves. This ultrasound-guided PENG block differs from other blocks, in that it targets the branch of the joint that innervates the anterior part of the hip and, if done properly, does not result in limb weakness (4). It is used in hip surgery as an alternative to other regional nerve blocks such as fascia iliaca compartment block or femoral nerve block (7). Several newly randomized controlled trials (RCTs) have been used to estimate the efficacy of PENG block in participants undergoing hip surgery. Pascarella et al. (8) reported that PENG block showed superior results in reducing opioid requirements and pain intensity after hip surgery. Comparatively, PENG block did not reduce opiate demand or the maximum postoperative pain score in participants who underwent hip surgery (9, 10).

In view of the inconsistent results of these RCTs, we carried out a systematic review and meta-analysis to identify the effects of PENG block, with a focus on the analgesic effects compared with other peripheral nerve blocks in participants after hip surgery.

## 2. Methods

This meta-analysis was planned and conducted according to the Preferred Reporting Items for Systematic Reviews and Meta-Analyses (PRISMA) checklist (11). The authors registered the protocol in the International Prospective Register of Systematic Reviews (Registration Number: CRD42022356496). The current study did not require ethics approval or informed consent since no patient information was collected.

### 2.1. Search strategy

We searched the electronic databases of PubMed, Embase, Web of Science, and Cochrane Library from the establishment of the database to 25 August 2022. The process of searching was systematically executed by two researchers (L.Y. and X.S.), independently, without language, publication year, journal, or region restrictions. The following search terms were used: (“Pericapsul^*^”) AND (“nerve block” OR “Block, Nerve” OR “Blocks, Nerve” OR “Nerve Blocks” OR “Nerve Blockade” OR “Blockade, Nerve” OR “Blockades, Nerve” OR “Nerve Blockades” OR “Chemical Neurolysis” OR “Chemical Neurolyses” OR “Neurolyses, Chemical” OR “Neurolysis, Chemical” OR “Chemodenervation” OR “Chemodenervations”). Appropriate adjustments were made when searching the database and if the full-text article was available. The search strategies for each database are included in the Supplementary Table 1.

### 2.2. Selection criteria

Any studies that met the following criteria were included: (1) RCTs; (2) studies that included adult patients with hip surgery; (3) studies that compared the effect of PENG block with that of other peripheral nerve blocks; and (4) studies that reported postoperative pain-related outcomes. Conversely, the following types of articles were excluded: (1) non-randomized studies; (2) studies that compared the effect of PENG block with sham block; and (3) studies in which numerical data related to postoperative analgesia were not available. When there were any differences of opinion between the two authors selecting the included studies, a senior author (H.L.) was involved and made the final decision.

### 2.3. Data extraction

Two authors (L.Y. and X.S.) independently examined and screened the final enrolled RCTs and collected the following data: first author, publication year, country, sample size, type of surgery, type of anesthesia, treatment of PENG block group, treatment of control group, and primary outcome. Medians, interquartile ranges, and ranges were approximated as means and standard deviations using the quantile estimation method and the Box-Cox method of McGrath et al. (12) as well as the method for unknown non-normal distributions approach of Cai et al. (13). When the two independent examiners did not agree, a third reviewer (H.L.) made the final decision.

### 2.4. Primary and secondary outcomes

The primary outcome was postoperative 24-h intravenous morphine equivalent doses, which was defined as the sum of analgesic drug conversion to intravenous morphine consumption over 24 h postoperatively. The secondary outcome included the time of the first request for rescue analgesia, the static and dynamic pain scores at 6 and 24 h after surgery, and the incidence of PONV. Pain score is defined as a participant's reported feeling of discomfort using an 11-point visual analog scale (VAS) or a numerical rating scale (NRS) (0: none, 10: extreme pain). PONV is defined as a discomfort sensation in which the participant has the impulsion to vomit or is forced to expel the stomach contents from the mouth (14).

### 2.5. Quality assessment and certainty of evidence assessment

Two authors (L.Y. and X. S.) independently evaluated the risk of bias and quality of evidence. The risk of bias was assessed by using the Cochrane Risk of Bias tool for randomized trials, based on seven aspects: random sequence generation, allocation concealment, blinding of participants, blinding of outcome assessors, incomplete outcome data, selective reporting, and other potential bias; risk levels were categorized as low, unclear, or high (15). The GRADEpro guideline development tool was used to evaluate the evidence quality of each outcome (16, 17). Intensity levels of evidence were categorized as high-quality, medium-quality, low-quality, or very low-quality evidence. Any differences in the evaluation process were resolved by a third senior author (H.L.).

### 2.6. Statistical analysis

Meta-analysis was performed using Review Manager 5.4 (Cochrane Collaboration). The data used were the mean differences (MD) and odds ratio (OR), presented as 95% confidence intervals (CI). When the *p*-value was < 0.05 and the 95% CI did not include 1 for OR and 0 for the MD, the difference was considered statistically significant. The chi-square test was used for heterogeneity analysis, and heterogeneity was assessed by *I*^2^. When the *I*^2^-values were < 25%, 25–50%, and >50%, the heterogeneity levels corresponded to low, medium, and high. A decision was made to use a random effect model due to the anticipated heterogeneity in this study. The sensitivity analyses were performed, removing one study at a time and combining the other studies, to assess whether a single study significantly affected the pooled results.

Data presented in the original literature were used as the primary source for extraction; when data were not shown, we contacted the authors for more information. As a final resort, when mean and SD values were not reported for an outcome [the postoperative 24-h intravenous morphine equivalent doses (18, 19) and the static and dynamic pain scores at 6 and 24 h after surgery (19, 20)], these values were imputed using the calculation methods of two statistical experts McGrath et al. (12) and Cai et al. (13).

### 2.7. Assessment of publication bias

Due to the small number of studies in each comparison (the number available for analysis was 3–6), we could not reliably assess the risk of publication bias. Therefore, the publication bias test was not performed in this study.

## 3. Results

### 3.1. Study selection

**Figure 1:**
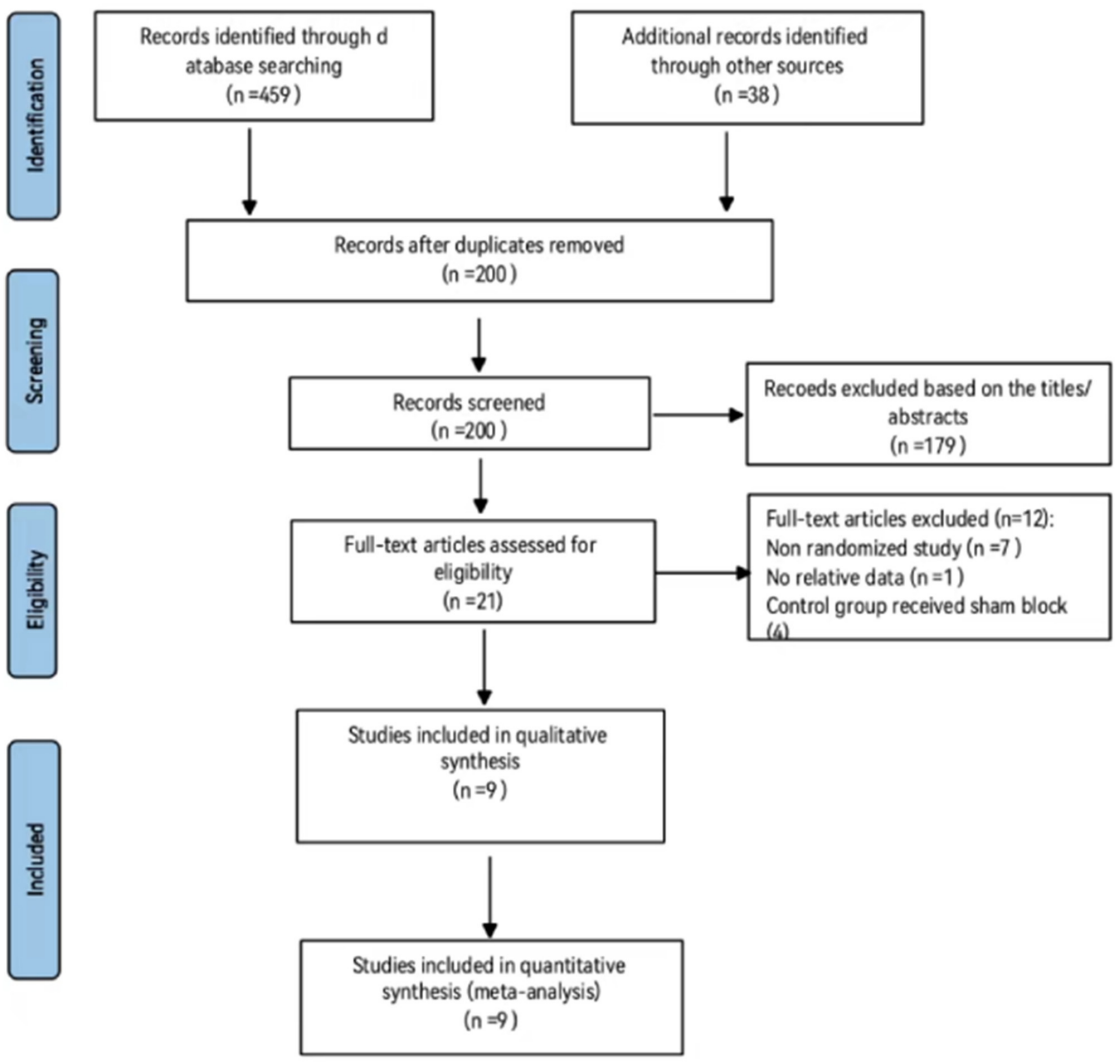
Flow diagram of included and excluded studies.

A total of 497 papers were found in the database search. Of these, 297 papers were duplicates; therefore, 200 papers were selected for this study. In addition, 146 (case report: 41; cohort study: 7; comment: 13; conference abstract: 10; protocol: 1; review: 26; withdrawal statement: 6; and letter: 42) and 33 (analgesia during postural changes: 7; anatomical research: 9; chronic pain treatment: 6; PENG block in other body parts: 9; hip arthroscope: 1; and combined with other block methods) papers were excluded using the titles and abstracts, respectively. The full text of the remaining 21 papers was reviewed and evaluated for eligibility. Out of which, 12 papers were excluded from the final analysis for the following reasons: non-randomized studies (*n* = 7), no relative data (*n* = 1), and when the effect of the PENG block was compared with the sham block (*n* = 4). Finally, nine RCTs were included in the meta-analysis (18–26) (Figure 1).

The characteristics of the included RCTs are shown in Table 1. Participants in one RCT underwent hip surgery under general anesthesia (21), seven RCTs enrolled those who were under spinal anesthesia (19, 20, 22–26), and one RCT enrolled patients who were under general anesthesia or spinal anesthesia (18). In the spinal anesthesia group, three RCTs (19, 20, 24) administered intrathecal opioids (fentanyl or morphine) combined with local anesthetics; however, the other two studies (23, 26) used only local anesthetics without adjuvants. The remaining studies did not describe the use of drugs for spinal anesthesia (18, 22, 25). The types of surgery were total hip arthroplasty (19–21, 23) and hip fracture surgery (18, 22, 24–26).

**Table 1:** Characteristics of included randomized controlled trials.

**References**	**Country**	**Number of patient (treatment/ control)**	**Type of surgery**	**Anesthesia method**	**Treatment of PENG group**	**Treatment of control group**	**Primary outcome**
Aliste et al. (17)	Chile	20/20	Total hip arthroplasty	Spinal anesthesia	20 mL of adrenalized 0.50% levobupivacaine	Fascia iliaca block: 40 mL of adrenalized 0.25% levobupivacaine	Incidence of quadriceps motor block 6 h after operation
Choi et al. (21)	Korea	27/27	Total hip arthroplasty	General anesthesia	20 mL of 0.2% ropivacaine with epinephrine 1:200,000	Fascia iliaca compartment block: 30 mL of 0.2% ropivacaine with epinephrine 1:200,000	Pain scores at rest and during movement 48 h after operation
Lin et al. (18)	Australia	30/30	Hip fracture surgery	Spinal anesthesia or general anesthesia	20 mL of 0.75% ropivacaine	Femoral nerve block: 20 mL of 0.75% ropivacaine	Pain score 4 h after operation
Mosaffa et al. (22)	Iran	30/22	Hip fracture surgery	Spinal anesthesia	3 mL/kg (a maximum of 40 mL) of 0.5% ropivacaine	Fascia iliaca compartment block: 3 mL/kg (a maximum of 40 mL) of 0.5% ropivacaine	Pain score 12 h after operation
Hua et al. (23)	China	24/24	Total hip arthroplasty	Spinal anesthesia	0.4% ropivacaine 20 mL	Fascia iliaca block: 0.4% ropivacaine 30 mL	Pain score 48 h after operation
Jadon et al. (24)	India	33/33	Hip fracture surgery	Spinal anesthesia	25 ml mixture of 0.25% bupivacaine and dexamethasone (8 mg)	Fascia iliaca compartment block: 25 ml mixture of 0.25% bupivacaine and dexamethasone (8 mg)	Pain scores at rest and during movement 30 min after the block
Zheng et al. (19)	Korea	25/27	Total hip arthroplasty	Spinal anesthesia	30 mL of 0.5% ropivacaine	Periarticular infiltration: ropivacaine [0.75% ropivacaine (20 mL)], ketorolac (60 mg), and epinephrine (1 g) were mixed with normal saline (total volume 100 mL)	Pain score 12 h after surgery at rest
Natrajan et al. (25)	India	12/12	Hip fracture surgery	Spinal anesthesia	20 mL of 0.5% ropivacaine	Fascia iliaca compartment block: 20 mL of 0.5% ropivacaine	Pain score 24 h after operation
Senthil et al. (26)	India	20/20	Hip fracture surgery	Spinal anesthesia	30 mL 0.25% Levobupivacaine and 4 mg dexamethasone	Fascia iliaca compartment block: 30 mL 0.25% Levobupivacaine and 4 mg dexamethasone	Pain score 24 h after operation

PENG, Pericapsular nerve group.

### 3.2. Risk of bias assessment

**Figure 2:**
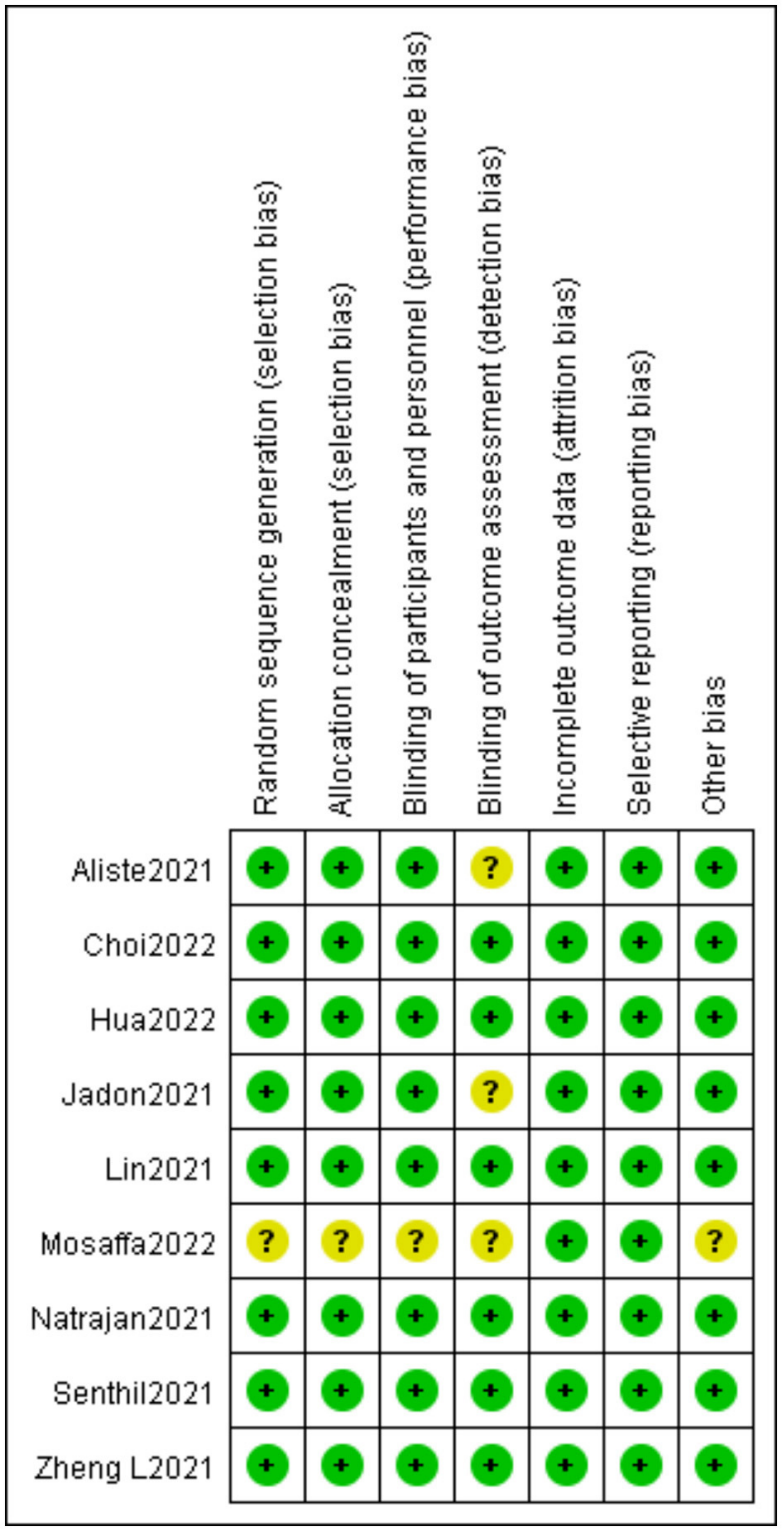
Risk of bias of included randomized controlled trials.

The risk of bias assessment results is shown in Figure 2. A low level of the overall risk of bias was observed for the included nine trials. Randomization of all patients into each group by appropriate methods was sufficient for allocation concealment in most studies. The two RCTs reviewed lacked sufficient details in the blinding outcome assessors, and in this case, we are conservative and thus inclined to classify the trial as “unclear risk of bias” (20, 24). Furthermore, when the random sequence generation, allocation concealment, blinding of participants and personnel, and blinding of outcome assessment were not described, we judged these bias evaluation items to be “unclear risk of bias” (22).

### 3.3. GRADE

The main results are shown in Table 2. When using the GRADE guidelines to assess the strength of the synthesized evidence, the evidence level of dynamic pain score 6 h after the operation, and the incidence of PONV were high. The certainty of the evidence was downgraded by high statistical heterogeneity and the results of various studies were inconsistent. Therefore, the other indicators obtained a moderate level of quality evaluation.

**Table 2:** Summary of findings including GRADE assessment.

**Outcomes**	**Number of patients (studies)**	**Effect**		**Certainty**	**Explanation**
		**Relative (95% CI)**	**Absolute (95% CI)**		
Postoperative 24 h morphine consumption	306 (6 RCTs)	-	MD 2.57 lower (5.42 lower to 0.27 higher)	⊕⊕⊕○ Moderate	The results of various studies are inconsistent with high heterogeneity
Time of the first request for rescue analgesia	142 (3 RCTs)	-	MD 1.79 higher (1.06 lower to 4.64 higher)	⊕⊕⊕○ Moderate	The results of various studies are inconsistent with high heterogeneity
The rate of PONV	170 (4 RCTs)	OR 1.29 (0.53 to 3.10)	36 more per 1,000 (from 65 fewer to 205 more)	⊕⊕⊕⊕ High	None
6 h static pain score	238 (5 RCTs)	-	MD 0.17 lower (0.92 lower to 0.57 higher)	⊕⊕⊕○ Moderate	The results of various studies are inconsistent with high heterogeneity
6 h dynamic pain score	134 (3 RCTs)	-	MD 0.69 lower (1.58 lower to 0.21 higher)	⊕⊕⊕⊕ High	None
24 h static pain score	186 (4 RCTs)	-	MD 0.25 lower (1.54 lower to 1.05 higher)	⊕⊕⊕○ Moderate	The results of various studies are inconsistent with high heterogeneity
24 h dynamic pain score	134 (3 RCTs)	-	MD 0.05 higher (0.84 lower to 0.93 higher)	⊕⊕⊕○ Moderate	High heterogeneity

CI, confidence interval; MD, mean difference; OR, odds ratio; RCTs, randomized controlled trials.

GRADE Working Group grades of evidence: The quality considers: (1) within study risk of bias (methodological quality); (2) the directness of the evidence; (3) heterogeneity of the data; (4) precision of effect estimates; and (5) risk of publication bias.

High certainty: We are very confident that the true effect lies close to that of the estimate of the effect. Moderate certainty: We are moderately confident in the effect estimate. The true effect is likely to be close to the estimate of the effect, but there is a possibility that it is substantially different. Low certainty: Our confidence in the effect estimate is limited. The true effect may be substantially different from the estimate of the effect. Very low certainty: We have very little confidence in the effect estimate. The true effect is likely to be substantially different from the estimate of effect.

### 3.4. Primary outcome

#### 3.4.1. Postoperative 24-h morphine consumption

**Figure 3:**
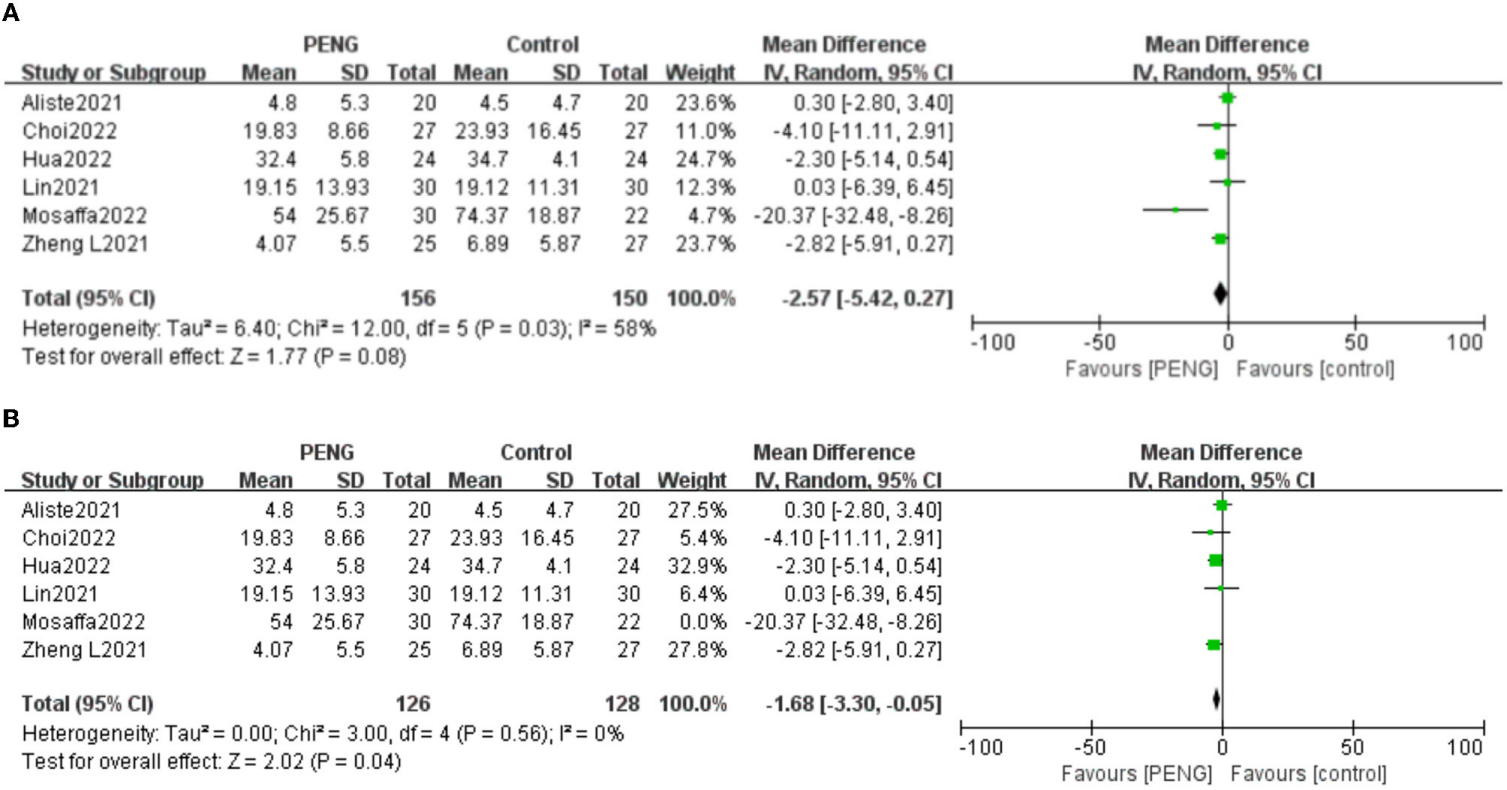
**(A)** Forest plot of postoperative 24 h morphine consumption. **(B)** Forest plot of postoperative 24 h morphine consumption after sensitivity analysis.

In total, six studies described postoperative 24-h morphine consumption (18–23). Overall, the PENG block group used 2.57 mg less morphine than the control group within 24 h after the hip operation, but this was not enough for a statistically significant difference [MD: −2.57, 95% CI (−5.42, 0.27), moderate quality evidence, *I*^2^ = 58%, *P* = 0.08] (Figure 3A).

Sensitivity analysis showed that when the study of Mosaffa et al. (22) was removed, the pooled results were reversed, and the heterogeneity was significantly reduced [MD: −1.68, 95% CI (−3.30, −0.05), *I*^2^ = 0%, *P* = 0.04] (Figure 3B). This shows that the study of Mosaffa et al. (22) is the main heterogeneity source and the result is unstable.

### 3.5. Secondary outcomes

#### 3.5.1. The time of the first request for rescue analgesia

**Figure 4:**
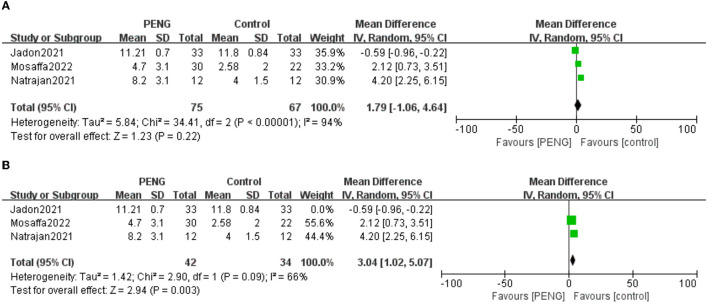
**(A)** Forest plot of the time of the first request for rescue analgesia. **(B)** Forest plot of the time of the first request for rescue analgesia after sensitivity analysis.

Three studies reported the time of the first request for rescue analgesia (22, 24, 25). There were no significant differences between the two groups [MD: 1.79, 95% CI (−1.06, 4.64), *I*^2^ = 94%, *P* = 0.22] (Figure 4A). The certainty of the evidence was evaluated as moderate.

The sensitivity analysis showed that the study of Jadon et al. (24) significantly affected the result and heterogeneity of the pooled analysis [MD: 3.04, 95% CI (1.02, 5.07), *I*^2^ = 66%, *P* = 0.003] (Figure 4B). Therefore, this indicated that it was an unstable outcome.

#### 3.5.2. Static pain scores 6 h after surgery

**Figure 5:**
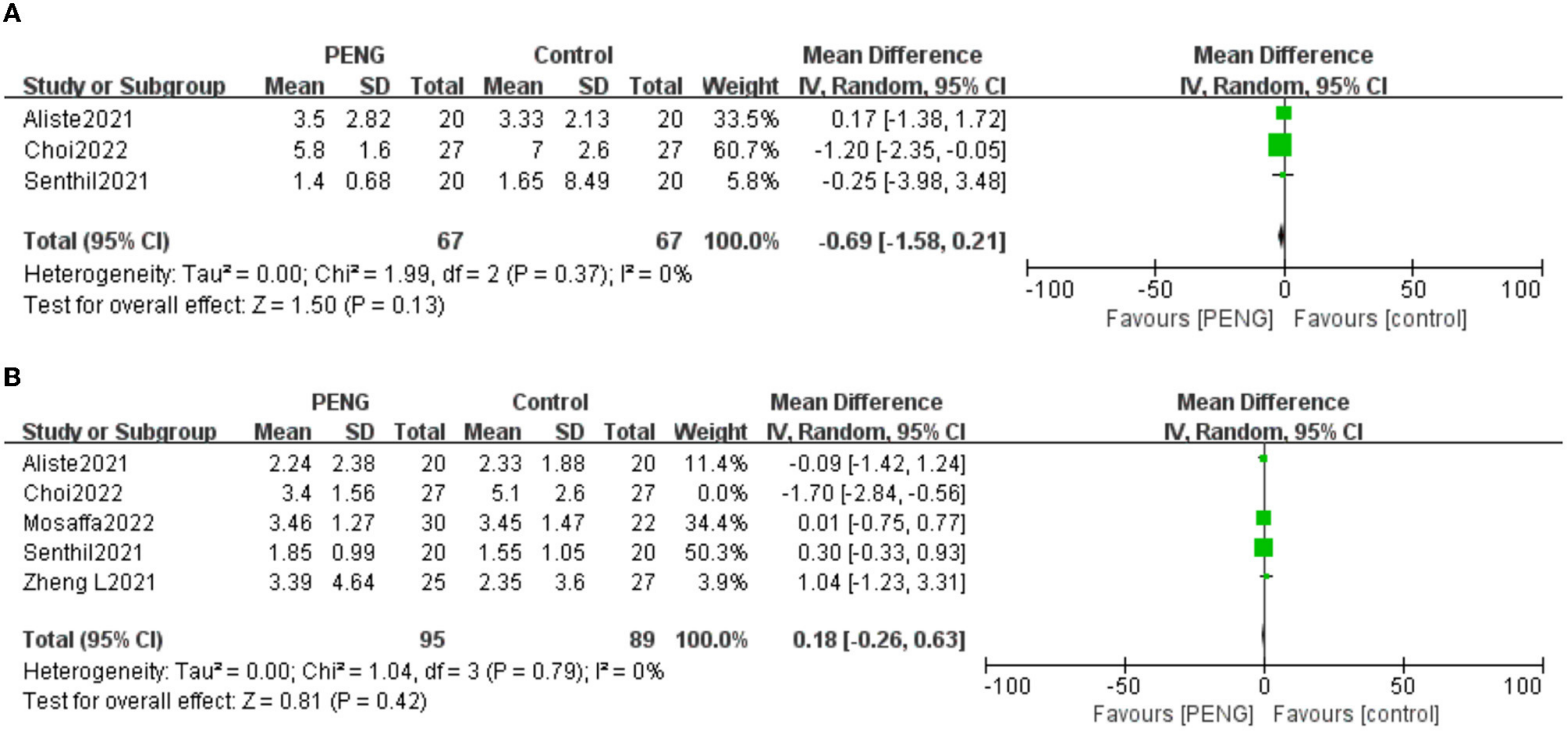
**(A)** Forest plot of the static pain scores 6 h postoperative. **(B)** Forest plot of the static pain scores 6 h postoperative after sensitivity analysis.

Participants included in the five trials (19–22, 26) were asked to grade the static pain scores 6 h postoperative. No significant differences were found between the two groups [MD: −0.17, 95% CI (−0.92, 0.57), moderate quality evidence, *I*^2^ = 60%, *P* = 0.65]. Subgroup analysis was done according to the different types of surgery. The subgroup analysis revealed a high degree of heterogeneity within the “total hip arthroplasty” subgroup [MD: −0.46, 95% CI (−1.96, 1.04), *I*^2^ = 67%, *P* = 0.55] (Figure 5A).

When examining the sources of heterogeneity, we carried out a sensitivity analysis. We found that the pooled analysis results remained unchanged after excluding the data of Choi et al. (21) but the heterogeneity decreased (MD = 0.18; 95% CI = −0.26 to 0.63; *P* = 0.42; *I*^2^ = 0%) (Figure 5B), indicating that this study is the main source of heterogeneity.

#### 3.5.3. Dynamic pain scores 6 h after surgery

**Figure 6:**
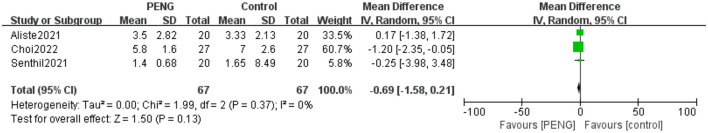
Forest plot of the dynamic pain scores 6 h after surgery.

Three studies reported dynamic pain scores 6 h after surgery (20, 21, 26). There was no significant difference between the two groups [MD: −0.69, 95% CI (−1.58, 0.21), high-quality evidence, *I*^2^ = 0%, *P* = 0.13] (Figure 6). We performed a sensitivity analysis and confirmed the stability of this result.

#### 3.5.4. Static pain scores 24 h after surgery

**Figure 7:**
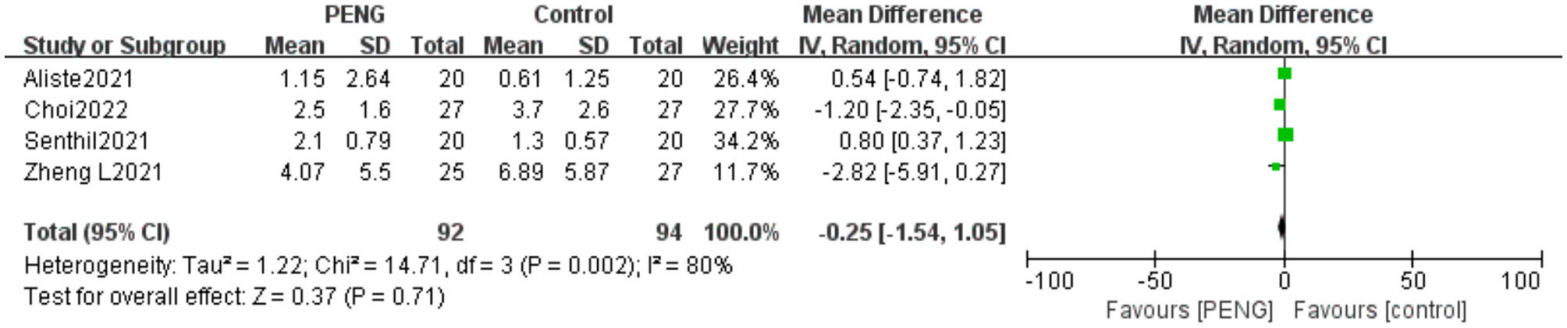
Forest plot of the static pain scores 24 h after surgery.

Four studies reported static pain scores 24 h after surgery (19–21, 26). There was no significant difference between the PENG block and the other nerve block techniques [MD: −0.25, 95% CI (−1.54, 1.05), moderate quality evidence, *I*^2^ = 80%, *P* = 0.71] (Figure 7).

We performed sensitivity analyses and found that the pooled analysis result is stable, and the heterogeneity was still high.

#### 3.5.5. Dynamic ***pain*** scores 24 h after surgery

**Figure 8:**
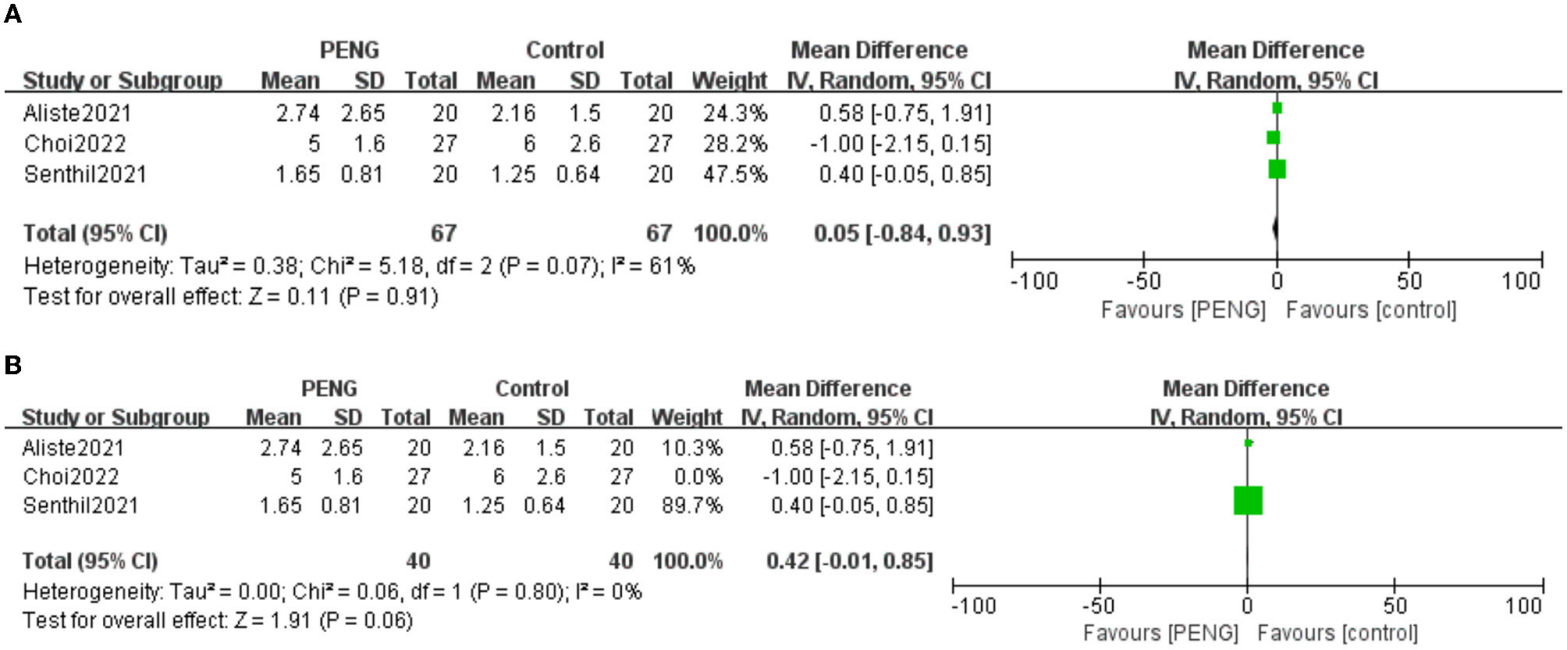
**(A)** Forest plot of the dynamic pain scores 24 h postoperative. **(B)** Forest plot of the dynamic pain scores 24 h postoperative after sensitivity analysis.

Three trials reported the dynamic pain scores 24 h after surgery (20, 21, 26). No significant differences were found between the two groups [MD: 0.05, 95% CI (−0.84, 0.93), moderate quality evidence, *I*^2^ = 61%, *P* = 0.91] (Figure 8A).

We carried out a sensitivity analysis and found that the pooled analysis results remained unchanged after excluding the data of Choi et al. (21), but the heterogeneity was low (MD = 0.42; 95% CI = −0.01 to 0.85; *P* = 0.06; *I*^2^ = 0%) (Figure 8B), indicating the main source of heterogeneity.

#### 3.5.6. Incidence of PONV

**Figure 9:**
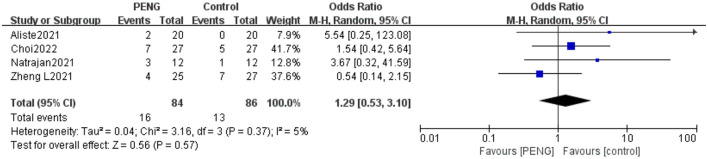
Forest plot of the incidence of PONV.

Four studies reported the incidence of PONV (19–21, 25). The pooled effect showed that the incidence of PONV was similar between the two groups (OR: 1.29, 95% CI = 0.53–3.10, high-quality evidence, *I*^2^ = 5%, *P* = 0.57) (Figure 9). The sensitivity analysis confirmed that the result is stable.

## 4. Discussion

During this meta-analysis and systematic review, we evaluated the role of PENG block in the effectiveness of postoperative analgesia after hip surgery. We did not find any significant difference in PENG block in postoperative pain-related indicators and PONV compared with other nerve block techniques. Statistically, PENG block seemed to be as effective as other types of nerve blocks for alleviating pain after hip surgery. We note that the pooled result of postoperative 24-h morphine consumption in published relevant meta-analysis is inconsistent with ours (27). Due to different perspectives and inclusion criteria, the included studies are not quite the same. The purpose of this study was to compare PENG block with other nerve block methods and exclude those studies with a control group that received a sham block.

Traditionally, regional nerve block in hip surgery is performed using femoral nerve or fascia iliaca block. While partially effective, these blockade methods lead to a decrease in muscle strength (28). In the present research, the quadriceps muscle strength was similar between the PENG block group and the sham block group after surgery, and the ranges of quadriceps muscle activity and “time to first walk” were even significantly better in the PENG group (8, 9, 29). The exercise retention effect enables patients to move early after surgery, which in itself is associated with fewer complications, shorter hospital stays, and lower mortality (30, 31). As a result, the PENG block is capable of moving as quickly and with less pain than patients in the sham group. Furthermore, several studies have reported that the PENG block also protects motor function better than the femoral nerve and fascia iliaca compartment block (18, 20, 26).

We performed a sensitivity analysis for each comparison; some pooled results were reversed, and the heterogeneity decreased significantly. The studies rejected by the sensitivity analysis were reviewed, and we found that the reasons for rejection included some bias risks that were unclear (22), the style of anesthesia was different from other studies and general anesthesia was chosen (21), and a lack of description of the blind methods from the evaluator of the results (24). However, the reversed results did not contradict our conclusion. It is worth noting that although the nerve block methods of the control group in the nine RCTs were different, the sensitivity analysis did not find that this would lead to heterogeneity and unsteadiness of the pooled results. We will continue to pay attention to the relevant research results in the future and go on to update the current meta-analysis.

Several limitations exist in the present study. First, because the number of RCTs is not yet adequate and the evaluation methods of muscle strength index vary greatly, data types include dichotomous variables and continuous variables. We do not perform a quantitative analysis comparing the PENG block to control groups in preventing quadriceps weakness. Second, in several measurements, moderate to high heterogeneity was observed. Due to the small number of RCTs included for each pooled outcome, only the static pain scores 6 h postoperative were successfully analyzed in subgroups. The results of the subgroup and sensitivity analyses together showed that the study by Choi et al. (21) was the main source of heterogeneity. The study administered general anesthesia differently from other studies, which may have contributed to its increased heterogeneity. Other pooled results that could not be analyzed by subgroups could only be analyzed by sensitivity analysis and pooled analysis using a random effects model.

More and more researchers believe that it is inappropriate to go back and re-select fixed effect or random effect models after knowing the size of the heterogeneity (32). Compared with the fixed effect model, the random effect model gives a more conservative pooled value and a broader inter-group variability, with an increasing preference for a random-effect analysis *a priori* (32). Third, sensitivity analysis showed that certain results were unstable, and the meta-analysis should be strengthened by further studies. Fourth, the number of studies included is small, and the sample size was 12–33 patients per group, which may increase the possibility of class I errors. Because of the small number of studies per comparison, we could not reliably assess the risk of publication bias. Therefore, no publication bias test was performed in this study. Fifth, for ethical reasons, RCTs apply other nerve block methods as a control group to compare with the PENG block. This meta-analysis combined analgesia-related data from multiple nerve block methods as a control group, and in doing so, there was a risk of elevated heterogeneity, although sensitivity analysis did not identify this as a source of high heterogeneity.

## 5. Conclusion

In summary, PENG block provides an effective analgesic, similar to other peripheral nerve blocks in hip surgery. Considering that PENG block can better preserve motor function, it can be used as a promising regional anesthetic technique to replace other nerve blocks in hip surgery. At the same time, we encourage more relevant research to update this meta-analysis.

## Data availability statement

The original contributions presented in the study are included in the article/Supplementary material, further inquiries can be directed to the corresponding author.

## Author contributions

LY conceived and designed the study. LY and XS performed the literature search and drafted the manuscript. HL was responsible for providing methodology and manuscript editing. All authors critically revised the manuscript and approved the final version for submission.

## Supplementary material

Supplementary Table S1 Search strategies for each database.
