# Supplementary material for: The efficacy of pericapsular nerve group block for postoperative analgesia in patients undergoing hip surgery: A systematic review and meta-analysis of randomized controlled trials
Source: Front Med (Lausanne). 2023 Feb 24;10:1084532. doi: 10.3389/fmed.2023.1084532 (PMC9998683; doi:10.3389/fmed.2023.1084532)
Supplement: Supplementary Table S1 — Search strategies for each database. [file Table_1.doc]

**Embase**

Session Results

.......................................................

No. Query Results Results Date

#17. ('nerve block'/exp OR 'brachial plexus 121 27 Aug 2022

anesthesia':ab,ti OR 'cervical plexus

block':ab,ti OR 'ganglion block':ab,ti OR

'intercostal nerve block':ab,ti OR 'lumbar plexus

block':ab,ti OR 'paracervical block':ab,ti OR

'quadratus lumborum block':ab,ti OR 'retrobulbar

anesthesia':ab,ti OR 'stellate ganglion

block':ab,ti OR 'transversus abdominis plane

block':ab,ti) AND 'pericapsul*':ab,ti

.......................................................

**Cochrane Library database Retrieval strategy**

#1 (nerve block) (Word variations have been searched)

#2 (Pericapsul*) (Word variations have been searched)

#3 MeSH descriptor: [Nerve Block] explode all trees

#4 #1OR#3

#5 #2AND#4

**pubmed Retrieval strategy**

((Pericapsul*[Title/Abstract]) AND ((((((((((((((nerve block[Title/Abstract]) OR (Block, Nerve[Title/Abstract])) OR (Blocks, Nerve[Title/Abstract])) OR (Nerve Blocks[Title/Abstract])) OR (Nerve Blockade[Title/Abstract])) OR (Blockade, Nerve[Title/Abstract])) OR (Blockades, Nerve[Title/Abstract])) OR (Nerve Blockades[Title/Abstract])) OR (Chemical Neurolysis[Title/Abstract])) OR (Chemical Neurolyses[Title/Abstract])) OR (Neurolyses, Chemical[Title/Abstract])) OR (Neurolysis, Chemical[Title/Abstract])) OR (Chemodenervation[Title/Abstract])) OR (Chemodenervations[Title/Abstract]))) AND (((((((((randomized controlled trial[Publication Type]) OR (controlled clinical trial[Publication Type])) OR (randomized[Title/Abstract])) OR (placebo[Title/Abstract])) OR (randomly[Title/Abstract])) OR (trial[Title/Abstract])) OR (groups[Title/Abstract])) OR (drug therapy[MeSH Subheading])) NOT ((animals[MeSH Terms]) NOT (humans[MeSH Terms])))
